# The effect of deprivation and the systemic inflammatory response on outcome following curative resection for colorectal cancer

**DOI:** 10.1038/sj.bjc.6601156

**Published:** 2003-08-12

**Authors:** D C McMillan, K Canna, C S McArdle

**Affiliations:** 1 University Department of Surgery, Royal Infirmary, Glasgow G31 ZER, UK

**Keywords:** colorectal cancer, deprivation, the systemic inflammatory response, C-reactive protein, survival

## Abstract

The presence of a systemic inflammatory response predicted cancer-specific (HR 2.55, 95% CI 1.22–5.32, *P*<0.05) and overall survival (HR 2.12, 95% CI 1.17–3.87, *P*<0.05), independent of Dukes stage, in patients who had undergone apparently curative surgery for colorectal cancer (*n*=158). Deprivation predicted overall survival (HR 1.26, 95% CI 1.04–1.51, *P*<0.05) independent of Dukes stage.

There is evidence that, in a variety of tumour types, deprivation is associated with poor outcome. For example, in colorectal cancer there is a 5% survival difference between the most and least affluent members of society (Coleman *et al*, 1999). The basis of the difference in outcome according to socioeconomic status has not been clearly defined. It has been suggested that deprived patients have less access to health care and as a consequence present with more advanced disease and receive less-effective treatment (Kogevinas and Porta, 1997; Coleman *et al*, 1999). Detailed information on stage at diagnosis, type of surgery and subsequent treatment was not included in the above analyses.

However, in a recent analysis of outcome in over 2000 patients undergoing surgery for colorectal cancer, there was no evidence that mode or stage at presentation, the proportion of patients undergoing curative resection or postoperative mortality accounted for the difference in survival between the affluent and deprived patients (Hole and McArdle, 2002). After adjustment for the above factors, there was a 36 and 26% excess risk of overall and cancer-specific death, respectively, in the most deprived following curative resection. Therefore, it would appear that neither stage of disease at presentation nor variations in treatment are responsible for the difference in survival between the affluent and deprived.

Disease progression is a complex process that depends on a series of interactions between tumour and host cells. It is not only the intrinsic properties of tumour cells that determine invasion and metastasis, but also the environment in which malignant cells find themselves (Balkwill and Mantovani, 2001). Therefore, an alternative hypothesis to explain the effect of deprivation on outcome following curative resection for colorectal cancer is that the tumour–host response in the most deprived patients is altered.

One aspect of the host response that has recently generated interest is the nonspecific systemic inflammatory response which is recognised to be associated with primary operable colorectal cancer. Indeed, there is evidence that the systemic inflammatory response (as evidenced by C-reactive protein) predicts recurrence (McMillan *et al*, 1995), overall (Nielsen *et al*, 2000; McMillan *et al*, 2003) and cancer-specific (McMillan *et al*, 2003) survival, independent of stage, in patients who have undergone curative resection for colorectal cancer.

The aim of the present study was to examine the relationship between deprivation, the systemic inflammatory response and survival in patients who had undergone curative resection for colorectal cancer.

## PATIENTS AND METHODS

One hundred and fifty-eight patients with histologically proven colorectal cancer who, on the basis of laparotomy findings and preoperative abdominal computed tomography, were considered to have undergone curative resection between 1993 and 1999 in a single surgical unit at Glasgow Royal Infirmary were included in the study. Prior to surgery, a blood sample was taken for the measurement of C-reactive protein. At this time no patient showed clinical evidence of tumour recurrence, infection or other inflammatory conditions. The tumours were staged using conventional Dukes' classification (Dukes and Bussey, 1958).

The extent of deprivation was defined using the Carstairs deprivation index (Carstairs and Morris, 1991). This is an area-based measure derived from the 1991 census, using the postcode of residence at diagnosis, which divides the score into a seven-point index. For illustrative purposes, the results are presented by amalgamating the seven categories into three groups: affluent (categories 1 and 2), intermediate (categories 3–5) and deprived (categories 6 and 7). The Carstairs deprivation index has been extensively utilised in cancer patients (Edwards and Jones, 1999) and is particularly appropriate for use in the central belt of Scotland (MacKie and Hole, 1996).

The study was approved by the Research Ethics Committee of the Royal Infirmary, Glasgow, UK.

C-reactive protein was measured by Fluorescence Polarisation Immunoassay using an Abbott TDX analyser and Abbott reagents (Abbott Laboratories, Abbott Park, IL, USA). The limit of detection of the assay is a C-reactive protein concentration of less than 5 mg l^−1^. The coefficient of variation, over the range of measurement, was less than 5% as established by routine quality control procedures.

### Statistics

Data are presented as median and range. Based on previous work, a C-reactive protein concentration of greater than 10 mg l^−1^ was considered to indicate the presence of a systemic inflammatory response.

Survival analysis was performed using the Cox proportional hazard model with patients' age, sex, tumour site, Dukes stage, deprivation index and C-reactive protein as prognostic variables. Deaths up to the end of June 2002 have been included in the analysis. Multivariate survival analysis was performed using a stepwise backward procedure to derive a final model of the variables that had a significant independent relationship with survival. To remove a variable from the model, the corresponding *P*-value had to be greater than 0.10. Analysis was performed using SPSS software (SPSS Inc., Chicago, IL, USA).

## RESULTS

Baseline characteristics of the patients (*n*=158) who underwent curative surgery for colorectal cancer are shown in Table 1

**Table 1:** Characteristics of patients who underwent curative resection for colorectal cancer

**Patients**	**(*n*=158)**
*Age group* (years)	
<65	45 (29%)
65–74	53 (33%)
75+	60 (38%)
*Sex*	
Male	80 (51%)
Female	78 (49%)
*Deprivation index*	
1, 2	4 (3%)
3, 4, 5	65 (41%)
6, 7	89 (56%)
*Tumour site*	
Colon	109 (69%)
Rectum	49 (31%)
*Dukes stage*	
B	99 (63%)
C	59 (37%)
*Adjuvant therapy*	22 (14%)

. Three percent of the patients were affluent and 56% were deprived. Twenty-two patients received adjuvant 5-fluorouracil-based chemotherapy.

The minimum follow-up was 30 months and the median follow-up was 49 months. During this period, 50 patients died, 32 patients of their cancer and 18 of intercurrent disease, and seven of cardiovascular disease.

*Cancer-specific survival*: On univariate analysis, age, Dukes stage and C-reactive protein concentration were predictors of cancer-specific survival (Table 2

**Table 2:** Relationship between variables and cancer-specific survival following curative resection for colorectal cancer

	**Univariate analysis**		**Multivariate analysis**	
	**Hazard ratio (95% CI)**	**(*P*-value)**	**Hazard ratio (95% CI)**	**(*P*-value)**
Age				
(<65/65–74/>75 years)	2.72 (1.62–4.56)	<0.001	2.58 (1.56–4.27)	<0.001
Sex (male/female)		0.506		0.685
Deprivation index (1–7)		0.420		0.354
Tumour site				
(colon/rectum)		0.311		0.281
Dukes stage (B/C)	2.28 (1.14–4.57)	0.019	2.57 (1.25–5.26)	0.010
Adjuvant therapy (yes/no)		0.803		0.941
C-reactive protein				
(⩽10/>10 mg l^−l^)	2.13 (1.05–4.32)	0.037	2.55 (1.22–5.32)	0.013

). On multivariate analysis, age (*P*<0.001), Dukes stage (*P*⩽0.01) and C-reactive protein concentration (*P*<0.05) retained independent significance.

*Overall survival*: On univariate analysis, age, deprivation, Dukes stage and C-reactive protein concentration were significant predictors of overall survival (Table 3

**Table 3:** Relationship between variables and overall survival following curative resection for colorectal cancer

	**Univariate analysis**		**Multivariate analysis**	
	**Hazard ratio (95% CI)**	**(*P*-value)**	**Hazard ratio (95% CI)**	**(*P*-value)**
Age				
(<65/65–74/>75 years)	2.84 (1.86–4.34)	<0.001	2.81 (1.83–4.29)	<0.001
Sex (male/female)		0.132		0.261
Deprivation index (1–7)	1.22 (1.02–1.47)	0.032	1.26 (1.04–1.51)	0.018
Tumour site				
(colon/rectum)		0.310		0.240
Dukes stage (B/C)	2.37((1.36–4.13)	0.002	2.64 (1.48–4.71)	0.001
Adjuvant therapy (yes/no)		0.414		0.610
C-reactive protein				
(⩽10/>10 mg l^−l^)	2.02 (1.15–3.58)	0.015	2.12 (1.17–3.87)	0.014

). On multivariate analysis, age (*P*<0.001), deprivation (*P*<0.05), Dukes stage (*P*⩽0.001) and C-reactive protein concentration (*P*<0.05) all retained independent significance.

## DISCUSSION

In the present study, we examined a homogenous group of patients who had undergone potentially curative resection. More than half of the patients belonged to the most deprived category. This is not surprising since eight of the 15 most deprived areas in the UK are in Glasgow (Gordon *et al*, 1999). As a result, the population served by Glasgow Royal Infirmary provide an ideal cohort of patients in whom to study the effect of deprivation.

On univariate and multivariate analyses, age, Dukes stage and the systemic inflammatory response were significant independent predictors of cancer-specific survival. This is consistent with the results of previous studies that indicate that the presence of a systemic inflammatory response is a significant predictor of cancer-specific survival, independent of Dukes stage, in patients undergoing apparently curative surgery for colorectal cancer. In contrast, deprivation failed to achieve statistical significance. This would suggest that the impact of deprivation on cancer-specific survival is less than that of the systemic inflammatory response.

The mechanism by which the presence of a systemic inflammatory response might influence cancer-specific survival is not clear. However, it is known that as part of the systemic inflammatory response to the tumour there is a release of proinflammatory cytokines and growth factors, some of which may promote tumour growth (Moldawer and Copeland, 1997) and hence influence survival. Furthermore, given that C-reactive protein concentrations are primarily determined by interleukin-6 concentrations, it is of interest that recent studies have shown that increased circulating concentrations of interleukin-6 in colorectal cancer are related to its production by the tumour (Kinoshita *et al*, 1999). It may therefore be that the presence of a systemic inflammatory response is a reflection of the malignant potential of the tumour.

On univariate and multivariate analysis, deprivation, Dukes stage and the systemic inflammatory response were significant independent predictors of overall survival. These results are therefore consistent with previous studies showing an association between deprivation and overall survival (Kogevinas and Porta, 1997; Hole and McArdle, 2002), and the presence of a systemic inflammatory response and overall survival (Nozoe *et al*, 1998; Nielsen *et al*., 2000) in patients undergoing apparently curative surgery for colorectal cancer.

It was of interest that deprivation achieved significance as a predictor of overall survival but not cancer-specific survival. This was because approximately one-third of deaths were due to intercurrent disease, most commonly cardiovascular disease. However, the mechanism by which deprivation impacts on overall survival is also not clear.

Although deprivation has been shown to be associated with increased comorbidity in cancer patients (Schrijvers *et al*, 1997), the relationship between deprivation, the presence of a systemic inflammatory response and death from intercurrent disease in patients with cancer remains to be established. However, obesity and smoking, which are associated with deprivation, are known to be associated with the presence of a systemic inflammatory response (Keonig *et al*, 1999). Furthermore, there is increasing evidence that the presence of a systemic inflammatory response is associated with increased risk of death from cardiovascular disease (Danesh *et al*, 2000).

In summary, the presence of the systemic inflammatory response contributes to poorer cancer-specific survival of patients who have undergone apparently curative surgery for colorectal cancer. Both deprivation and the presence of a systemic inflammatory response appear to contribute to overall survival.
